# Cellulose nanocrystal assisted trace silver nitrate to synthesize green silver nanocomposites with antibacterial activity†

**DOI:** 10.1039/d0ra07198f

**Published:** 2021-01-19

**Authors:** Jiang Zhu, Tao Tang, Chun-Yan Hu, Wen-Cai Xiang, Zhi-Qiang Chen, Liu Luo, He-Shan Yang, Hong-Pan Liu

**Affiliations:** Chongqing Key Laboratory of Environmental Materials & Remediation Technologies, Chongqing University of Arts and Sciences Yongchuan 402160 China jiangzhu415@cqwu.edu.cn; College of Chemistry and Environmental Engineering, Chongqing University of Arts and Sciences Yongchuan 402160 China

## Abstract

Cellulose nanocrystals (CNCs) with silver nanoparticles (AgNPs) are used for applications ranging from chemical catalysis to environmental remediation, and generation of smart electronics and biological medicine such as antibacterial agents. To reduce the synthesis cost of AgNPs and environmental pollution, microwave-assisted generation of AgNPs on the CNC surface (AgNPs@CNC) has been found to be useful, because microwave reaction has the advantages of simple reaction conditions, short reaction time and high reaction efficiency. The silver ions (Ag^+^) could be added to the CNC suspension and placed in the microwave reactor for a few minutes to produce AgNPs. AgNP generation was affected by factors such as the concentrations of Ag^+^ and CNC, and the power of the microwave, as well as the time of reaction. In this study, we used trace amounts of AgNO_3_ to rapidly synthesize AgNPs using a green microwave-based method instead of *Tollen's* reagent, and the antibacterial activity of the T1 sample showed that only using 0.03 mM (∼0.01 wt%) AgNO_3_ to synthesize AgNPs@CNC could achieve good antibacterial properties.

## Abstract 2

Cellulose nanocrystals (CNCs) with silver nanoparticles (AgNPs) are used for applications ranging from chemical catalysis to environmental remediation, and generation of smart electronics and biological medicine such as antibacterial agents.

## Graphical abstract

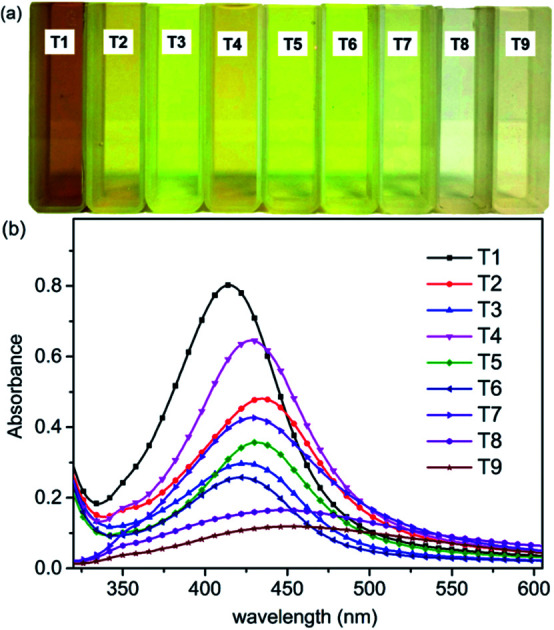

## 1 Introduction

Cellulose nanocrystals (CNCs) are natural and biodegradable rodlike nanoparticles processed from cellulose.^1–4^ As a promising new material, CNCs possess some unique properties, such as large surface area, unique optical character, and high mechanical strength.^5^ Furthermore, CNCs are commercially produced nanomaterials and their surfaces are easily chemically modified,^6–8^ promoting applications in real-life products and services.^9^ At the same time, they possess abundant hydroxyl groups, which assist in their dispersion in water, and charged surfaces.^4^ Owing to these specific physicochemical properties, CNCs can be widely used in scientific research, biological medicine and industrial production, such as drug delivery, bioimaging, tissue engineering,^7,10–16^ catalysis of chemical reactions,^17^ enhancement of functional materials,^18–21^ and the manufacture of luminescent materials.^22–25^ It is remarkable that CNCs can also be used as carriers for many metallic nanoparticles to promote the wide application of metallic nanoparticles.^26–31^

Antibacterial agents play an important role in eradicating bacteria and protecting human health.^32^ In particular, pathogenic *Escherichia coli* (*E. coli*) and *Staphylococcus aureus* (*S. aureus*) can cause health-threatening diseases such as clinical mastitis,^33^ dysentery^34^ and diarrhoea.^35^ Developing a stable, efficient and low-cost antibacterial agent is an urgent problem to be solved. AgNPs are the most common commercial antimicrobial nanomaterials.^36–38^ Although the antibacterial mechanism of AgNPs are not fully understood, several articles have reported that AgNPs adsorbed on the cell membrane to change its permeability and disturb its normal physiological functions.^39–41^ AgNPs are among the most valuable nanoparticles with superior properties, such as high antibacterial activity, which renders them suitable for using in tissue scaffolds, wound dressings, and protective clothing.^26^ In the past, AgNPs were synthesized by carrying on the surface of many materials, such as chitosan,^42–44^ kappa-carrageenan,^45^ cyclodextrin^31^ and graphene.^46,47^ As for the reduction of AgNPs on the surface of these materials, it is insufficient to widen the application of AgNPs that the materials cannot reach the nanometer scale, that the reduction process is expensive, that their dispersion in water is poor. However, CNC can facilitate the nucleation of metal nanoparticles from metallic salts with the assistance of reducing agents, while preventing the aggregation of metallic nanoparticles, to reach a narrow size distribution. For instance, Ag^+^ can be reduced to AgNPs using sodium borohydride (NaBH_4_) on the surface of CNC.^18,48–51^ In addition, glucose was applied as the reducing agent and CNC as the substrate to synthesize AgNPs from *Tollen's* reagent (Ag(NH_3_)_2_OH).^9^ Although the synthesis of antimicrobial AgNPs using these methods has been successful, it still poses certain restrictions on the end product. The raw materials used in these methods are toxic or expensive, and they cannot be applied for the development of green and economic methods for synthesis of AgNPs.

Recently, microwave reaction instruments were widely used to assist in the synthesis of AgNPs, and the synthesized AgNPs had a high antibacterial activity.^52–55^ Therefore, most studies have considered how to ameliorate the antibacterial activity of AgNPs as well as abate the addition amount of AgNPs. In this study, we used glucose as the reducing agent and CNC as the carrier to synthesize AgNPs in a microwave. The AgNPs prepared under different reaction conditions were characterized using UV/visible spectrometry. The AgNPs generated on the surface of CNC using the microwave reaction can be utilized to produce green and inexpensive AgNPs. This method of synthesizing AgNPs is simple and only requires trace amounts of silver nitrate, which can be further developed for large-scale commercial production of AgNPs.

## 2 Experimental

### 2.1. Materials

Cellulose microcrystalline (CMC) and silver nitrate (AgNO_3_, AR) were manufactured by Sinopharm Chemical Reagent Co., Ltd. (Shanghai, China). d-Glucose monohydrate (C_6_H_12_O_6_·H_2_O, AR) and sodium hydroxide (NaOH, AR) were produced by Chengdu Kelong Chemical Reagent Factory (Chengdu, China). All the reagents were used without further purification.

### 2.2. Preparation of CNC

H_2_SO_4_ solution was used to hydrolyze CMC during the preparation of CNC.^56^ First, 12.5 g of CMC was added to 64 wt% H_2_SO_4_ (200 mL) and the mixture was stirred at 50 °C for 4 h. Fivefold volume deionized water was then applied to the mixture to stop the reaction. The suspension was centrifuged to obtain crystals, which were washed in deionized water, centrifuged and separated again. This step was repeated five times for sample. Dialyzed against deionized water for several days until the water pH reached a value of 6.0–7.0. Finally, sample was freeze-dried for 24 h and stored in vacuum until use. In this experiment, the morphology of the CNC was characterized by a TEM with carbon-coated copper support grids (Fig. S1†), and the length of CNC was maintained at approximately 100 nm to 300 nm.

### 2.3. Optimization of AgNPs generation by orthogonal assay design

An orthogonal assay (Table 1) was designed to research the effects of CNC, silver ions, ultrasonic power, and reaction time on preparation of AgNPs. This orthogonal assay was used to find the optimized condition for production of AgNPs. In this experiment, AgNO_3_, NaOH and glucose (molar ratio 1 : 1 : 2) were used raw materials to synthesize AgNPs in 50 mL CNC dispersion.

**Table 1:** L9 (3^4^) Design of Orthogonal Array

#	CNC (mg mL^−1^)	Ag^+^ × 10^−2^ (mM)	Power (W)	Time (s)	UV/vis absorbance at 410 nm
T1	1.5	3.00	320	200	0.80
T2	1.5	1.50	240	250	0.39
T3	1.5	0.75	160	300	0.28
T4	0.5	3.00	240	300	0.56
T5	0.5	1.50	160	200	0.29
T6	0.5	0.75	320	250	0.24
T7	0	3.00	160	250	0.38
T8	0	1.50	320	300	0.14
T9	0	0.75	240	200	0.10

The reduction reaction was carried out by in a microwave oven (MCR-3, Gongyi yuhua instrument Co., Ltd, China) at different power and time. After reaction, the products were dialyzed against Milli-Q water in dark for 4 days at room temperature.

### 2.4. Characterization

UV-vis absorption spectra of AgNPs@CNC was taken over the wavelength range 200 to 800 nm using UV-vis spectrophotometer model UV-5500 (Shanghai METASH, China).

Morphology of AgNPs@CNC was observed on a GeminiSEM 300 (ZEISS, Germany) scanning electron microscope (SEM), and AgNPs@CNC was analyzed by energy dispersive spectrometer (EDS).

X-ray diffraction (XRD) patterns of AgNPs@CNC samples were measured by a DX-1000 diffractometer (Dandong Tongda Instrument Co., Ltd, China) using a CuKα radiation. The voltage and current were set to 30 kV and 20 mA, respectively. Diffraction patterns were recorded for 2*θ* values ranging from 5° to 80° at a scanning rate of 0.04° s^−1^.

FTIR spectra of the dried CNC and AgNPs@CNC samples were reported on a L1600400 Spectrum TWO DTGS spectrometer (Made in Liantrisant, UK) in the region of 4000−400 cm^−1^.

The X-ray photoelectron spectra of AgNPs@CNC were reported with an X-ray photoelectron spectrometer (XPS, Thermo Scientific). XPS was used by methods of a flood gun charge neutralizer system equipped with an Al Kα X-ray source (*hν* = 1486.6 eV).

The zeta potentials of AgNPs@CNC were measured using a dynamic light scattering (DLS) by 90plus PALS model (Brookhaven, USA). All measurements were implemented in triplicate.

Thermogravimetric analyses (TGA) were performed using NETZSCH STA449F3 thermogravimetric analyzer (Netzsch-Gerätebau GmbH, Selb, Germany). Materials were heated from 25 to 600 °C using a heating rate of 10 °C min^−1^ under a nitrogen flow (50 mL min^−1^).

### 2.5. Antibacterial tests

The antibacterial activity of the orthogonal array samples (T1–T9) were tested inhibition Gram-negative bacteria *E. coli* and Gram-positive bacteria *S. aureus* using the agar-diffusion approach. Bacteria were grown in the Luria−Bertani (LB) liquid medium (5 g L^−1^ casein tryptone, 2.5 g L^−1^ yeast extract, 5 g L^−1^ NaCl, and 500 mL deionized water, pH 7) at 37 °C for 24 h on a shaker bed at 200 rpm. The bacteria 100 μL were (∼10^8^ cfu mL^−1^ of *E. coli* and *S. aureus*) coated on the Luria–Bertani (LB) agar plates, and then 150 μL of orthogonal array samples were deposited and in wells (diameter = 7 mm) formed in the agar plates, and those plates were cultured at 37 °C for 24 h. Then, the ranges of the inhibition zone (*i.e.*, transparent area) were measured using a vernier caliper. All AgNPs@CNC samples were measured in triplicates.

Using the standard dilution method which determined the minimum inhibitory concentration (MIC) of T1 sample, MIC is defined as the minimum [Ag^+^] at which no bacterial turbid is viewed in all three parallel experiments after coculturing bacteria with AgNPs/CNC for 24 h, in this research. The T1 sample of Ag^+^ concentration was examined with inductively coupled plasma optical emission spectroscopy (ICP-OES, Perkin-Elmer, Optima 5300DV, USA). *E. coli* and *S. aureus* were cultured in the Luria-Bertani (LB) medium (10 g L^−1^ casein tryptone, 5 g L^−1^ yeast extract, and 10 g L^−1^ NaCl, pH 7) at 37 °C on a shaker bed at 200 rpm. Ag^+^ of different concentrations was prepared in each germfree test tube and inoculated with 4.8 × 10^6^ CFU mL^−1^ bacteria suspension for 24 h.

## 3 Results and discussion

### 3.1. Microwave-assisted generation of AgNPs

**Fig. 1:**
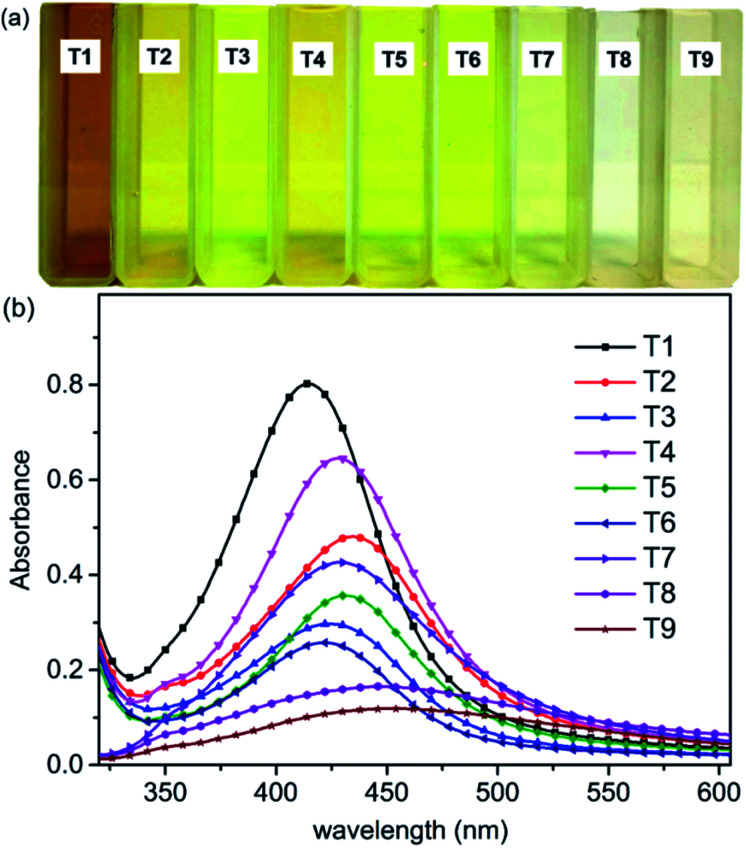
(a) Nanoparticle suspensions and (b) UV spectra of AgNPs@CNC (T1–T9).

Microwave reaction instruments could provide high energy in a very short time. Ag^+^ was added to the CNC suspension and placed in the microwave reactor for a few minutes to produce AgNPs. Images of the AgNPs@CNC suspensions prepared according to the orthogonal assay (T1–T9) described above are presented (Fig. 1a). The formation of light yellow AgNPs@CNC of nanoparticles was obvious from visual inspection of the reagents following microwave reaction.^57^ The color of AgNPs varies with the conditions of the orthogonal assay. The synthesis of AgNPs was confirmed by UV-vis spectrophotometry. UV absorption spectral intensity was chosen as an indicator for the selection of optimal conditions. Fig. 1b showed an absorbance spectrum with a peak at approximately 420 nm, indicating the formation of AgNPs, because the color and absorption spectra of samples at 420 nm is attributed to the excitation of surface plasmon vibrations of Ag atoms.^58,59^

We selected the optimal orthogonal assay conditions for synthetic AgNPs based on the intensity of UV-vis absorbance. The extreme range analysis of the intensity of UV absorption peak at 420 nm (Table S1†) indicated that the optimal AgNPs synthesis conditions were T1. Therefore, T1 sample was selected as the main characterization sample. All samples were freeze dried for 24 h and stored in vacuum before use. The samples (T7–T9) did not perform the next characterization because the absorbance of these samples was weak and the amount of sample after drying was very small.

### 3.2. Morphology of AgNPs@CNC

**Fig. 2:**
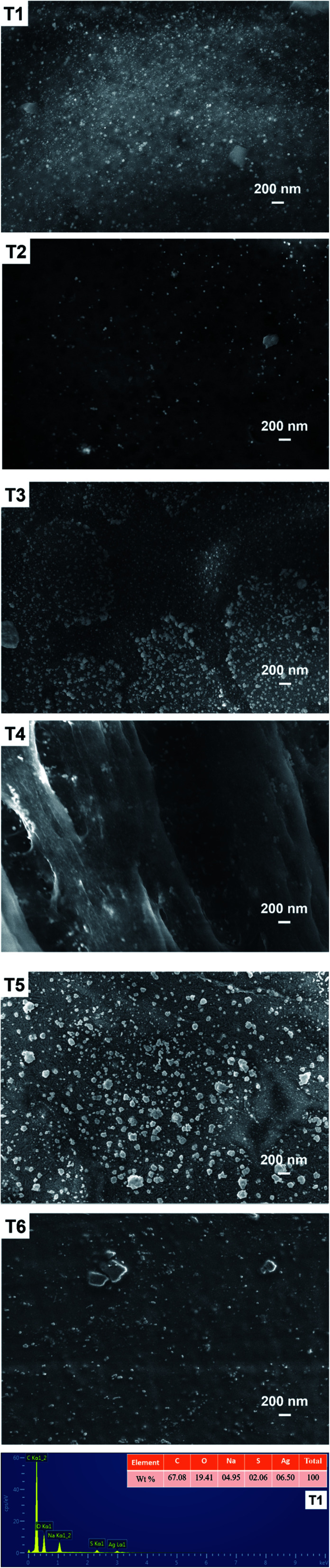
FE-SEM images of AgNPs@CNC (T1–T6) and the EDS curve for T1.

The surfaces of CNC had largely dense little light spots in the SEM of the T1 sample (Fig. 2). Similarly, the SEM image of the samples (T2–T4) obviously showed the presence of little spots on the surface, but the small spots of the samples is rarely relative to T1 sample. Meanwhile, it was not obvious that little light spots were observed in the samples (T5–T6). And the T5–T6 samples could only see the agglomerated CNC after drying. In the above analysis, it could be roughly considered that the small light spots were AgPNs, because the number of spots in the image of the SEM and the intensity of the UV-vis absorbance peak were mutually confirmed. In addition, the diameter of AgNPs was very small on the surface of CNC. This showed that AgNPs was not easily observed at the submicron scale. Fig. 2 shown the EDS spectrum for the T1 sample. The peaks of silver in the EDS images confirmed that the adsorption of AgNPs took place on the surface of CNC. In addition to the characteristic peaks of AgNPs, other peaks for primary elements, *i.e.*, C, O, Na, and S, were also exhibited. The display of these additional peaks in the EDS spectra were mainly due to the composition of the CNC and media containing these essential elements.

### 3.3. X-ray diffraction (XRD) analysis

**Fig. 3:**
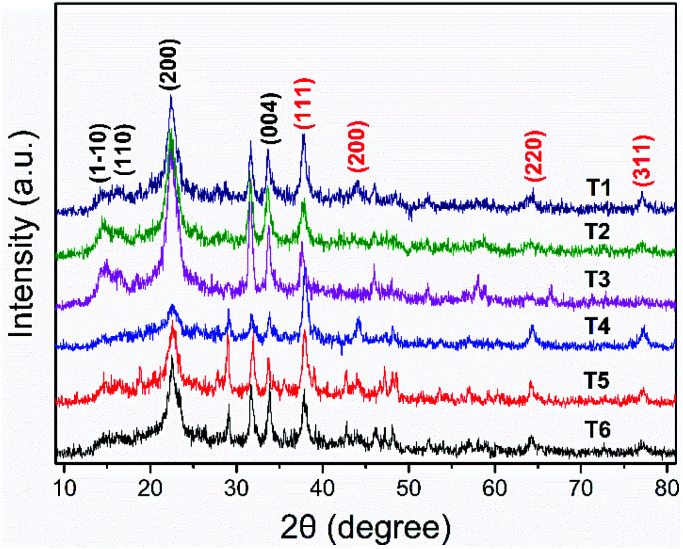
Wide-angle XRD patterns of the samples (T1–T6).

The XRD was used to explain and characterize the structure of the AgNPs generation (Fig. 3). Because the crystallization peak intensity of pure CNC (Fig. S2†) is too high, it was difficult to view the crystallization peak of Ag on the figure of the XRD curve of pure CNC and the curve of the orthogonal array samples. Therefore, we only gave the representative XRD patterns of AgNPs@CNC prepared from orthogonal assay samples (T1–T6). All samples revealed four typical diffraction peaks at about 2*θ* = 14.5°, 2*θ* = 16.5°, 2*θ* = 22.6° and 2*θ* = 34.0°, which were attributed to the presence of CNC, corresponding to (1–10), (110), (200) and (004) planes, respectively.^60,61^ Additional other small peaks from 30° to 80° in curves (T1–T6) were assigned to diffractions from AgNPs. The diffraction peaks from the AgNPs@CNC at about 38.1°, 44.4°, 64.4° and 77.5°, consistent with the crystalline planes of (111), (200), (220) and (311), respectively.^62^ There was a face-centered cubic (fcc) structure confirmed of crystalline AgNPs, according to the existence of these crystallization peaks.^63^ XRD pattern analysis was used to further illustrate the AgNPs on the surface of CNC under the trace silver ion concentration, because the XRD pattern exhibited extremely small characteristic peaks of AgNPs.

### 3.4. Chemical structure characterization of AgNPs@CNC

**Fig. 4:**
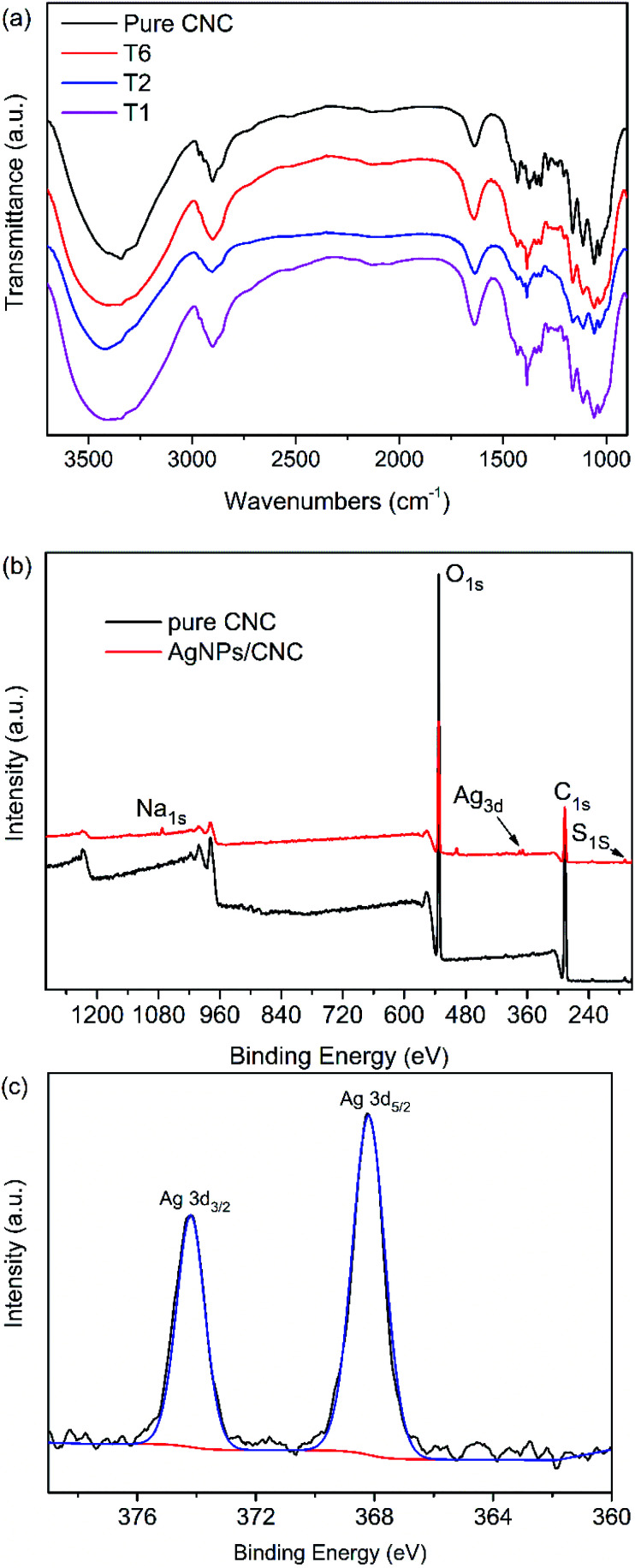
(a) FT-IR spectra of the pure CNC, T1, T2 and T6. (b) XPS spectra of the pure CNC and T1. (c) High resolution Ag 3d spectrum of the AgNPs@CNC.

The FTIR spectra of pure CNC and AgNPs@CNC were almost the same with each other (Fig. 4a). The main peak was OH stretching band at 3000−3700 cm^−1^ and CH stretching band at 2800−3000 cm^−1^. The peak located at 1633 cm^−1^ could be attributed to the absorbed moisture in the CNC. There were many weak strength peaks at 1250−1500 cm^−1^ and the explanation of these peaks need further studies. In conclusion, AgNPs@CNC did not form a new peak or the disappearance of the old peak compared with the pure CNC. This might be because the AgNPs were merely physically adsorbed and did not form chemical bonds on the CNC surface.

To further identify the synthesized AgNPs, element analyses of the sample were carried out by XPS. XPS survey of the pure CNC exhibited the peaks for C 1s (∼286 eV), O 1s (∼532 eV) and S 1s (∼169 eV). In addition, XPS survey of the AgNPs@CNC revealed the peaks for Na 1s (∼1072 eV), C 1s (∼286 eV), O 1s (∼532 eV) and S 1s (∼169 eV), and then AgNPs deposition on the CNC was confirmed by the appearance of Ag 3d doublet near 370 eV as shown in Fig. 4b. The high resolution Ag 3d spectrum exhibited in Fig. 4c was composed of two different peaks with binding energies of Ag 3d_3/2_ and Ag 3d_5/2_ at 368.2 and 374.2 eV, respectively, both ascribed to the Ag^0^.^29^ These peaks located at lower binding energies should not be appointed to oxidised silver compounds, because Ag 3d in oxides were located at more powerful binding energies, *e.g.* Ag 3d_5/2_ = 367.7 eV for Ag_2_O and 367.4 eV for AgO.^64^ These peaks also cannot be ascribed to the structure of Ag-C_5/2_ (∼366 eV) and Ag-C_3/2_ (∼372 eV) components.^65,66^ Moreover, the 6 eV dividing of the 3d doublet due to the spin−orbit coupling authorized the presence of Ag^0^ state on the CNC surface.^67,68^

### 3.5. Zeta potential analysis of the stability of AgNPs@CNC suspension

**Fig. 5:**
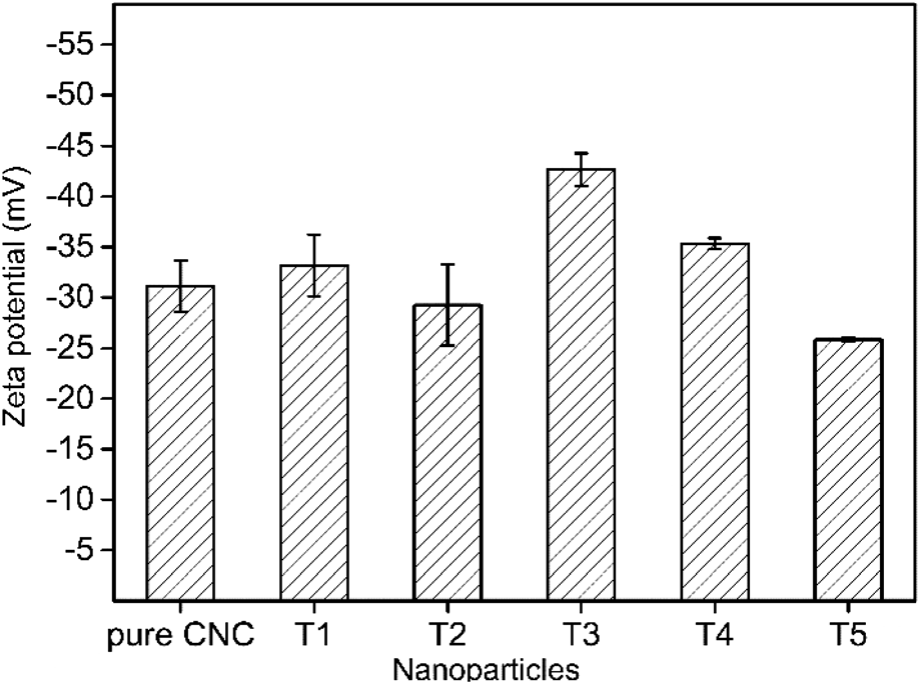
Zeta potential of AgNPs stabilized by CNC.

The zeta potential was a considerable parameter for determining the stability of AgNPs@CNC suspensions. For a physically stable nanoparticle suspension to be stabilized solely by electrostatic repulsion, a zeta potential of ±30 mV was required as a minimum.^69^ Zeta potential data of the AgNPs@CNC were exhibited in Fig. 5. These showed that the AgNPs@CNC had a high stability of the suspension, because samples potential values were >30 mV except the T2. Although the zeta potential value of the T1 sample was not the highest among all samples, it was about 33.16 ± 3.06 mV that was 10.5% higher than 30 mV. In addition, the zeta potential value of the T1 sample was higher than the value 31.13 ± 2.54 mV of pure CNC suspension. This indicated that controlling the appropriate amount of reduction of AgNPs on the surface of CNC did not affect the dispersion of CNC in deionized water, and T1 samples could be as stable as pure CNC suspensions.

### 3.6. Thermal stability analysis of AgNPs@CNC

**Fig. 6:**
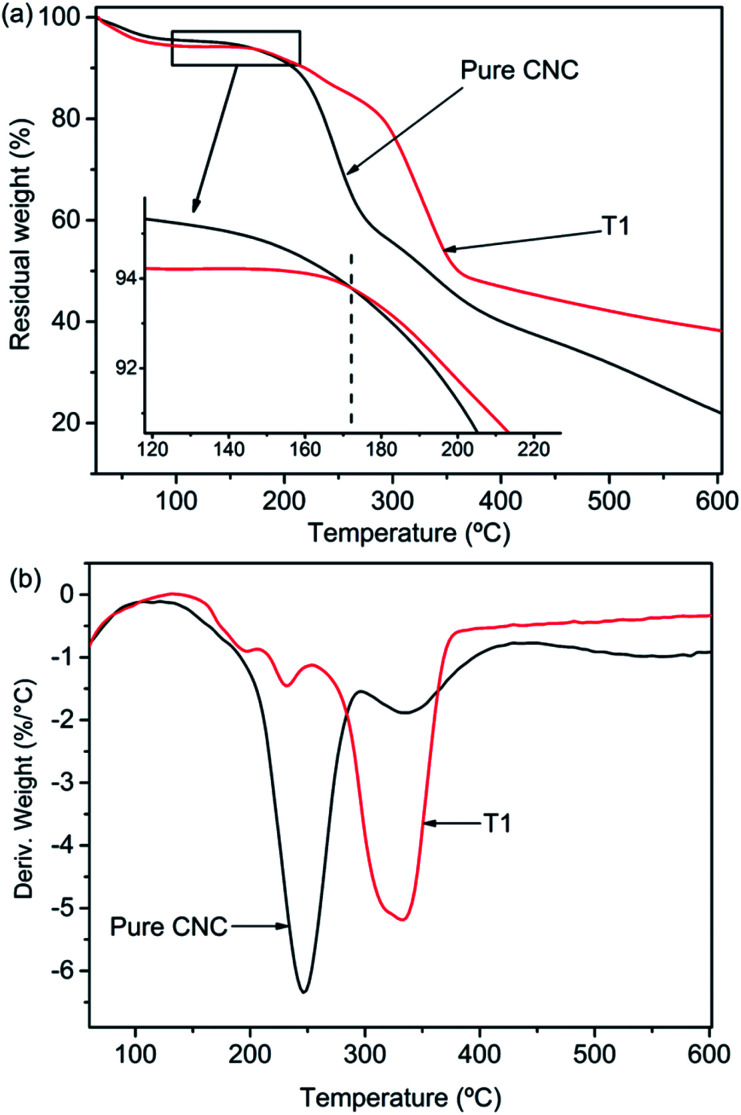
(a) TGA and (b) DTG thermograms of AgNPs@CNC.

Fig. 6a shown the TGA curves of pure CNC and the sample of T1. But the first mass loss of the materials was the same, two samples revealed different thermal degradation processes. The first mass loss in the range 50–100 °C was ascribed to the vaporization of water loosely bound to the materials. The next degradation procedure in the range of 130 °C to 400 °C was related to the dehydration and depolymerisation processes, followed by the generation of char residue at temperatures above 400 °C. The intermediate thermal degeneration of the CNC was generally associated with the formation of crosslinked structures from the radical products, which leads to the generation of thermally steady materials that were included in the char residue. The temperature associated with 50% mass reduction of neat CNC and AgNPs@CNC were ∼331 °C and ∼358 °C respectively, and the maximum thermal decomposition temperatures (*T*_max_ shown in Fig. 5b) of pure CNC and AgNPs@CNC were ∼246 °C and ∼333 °C, respectively. This could be ascribed to the existence of the AgNPs on the CNC surface, which reinforced the thermal stability of the CNC. The AgNPs reduced the migration of the CNC chains which inhibited the transfer of free radicals, thereby limiting the interchain reaction so that the degradation of the CNC occurred at higher temperatures.^68^ It was also possible that small and dense AgNPs interacted with the CNC surface to delay further decomposition of the material.

### 3.7. Antimicrobial activity

**Fig. 7:**
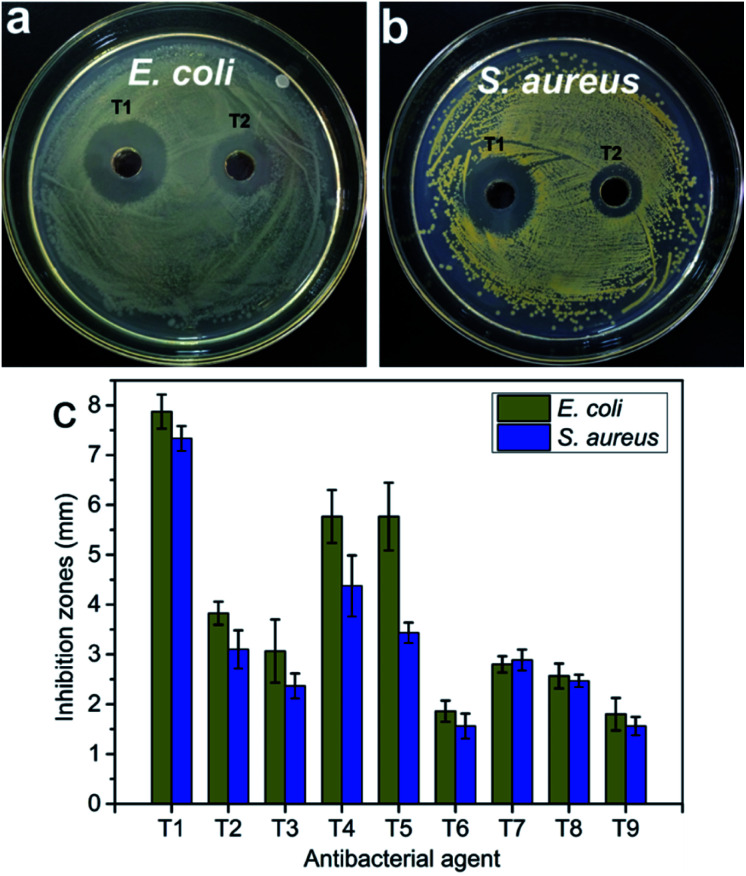
The photos of the antibacterial activity of AgNPs@CNC samples (a, b) and (c) the inhibition zones measured by using the vernier caliper.

In the antibacterial activity test, the different concentrations of pure CNC suspension (*i.e.*, 0.5 mg mL^−1^ and 1.5 mg mL^−1^) and deionized water were selected as controls. No antibacterial activity was observed for pure CNC suspension and deionized water (Fig. S3†). However, the incorporation of the AgNPs into the CNC surface caused antibacterial activity, and the CNC suspension exhibited significant inhibition against bacterial growth (Fig. 7). Because pure CNC suspension and deionized water did not have any antibacterial activity, the antibacterial activity of the AgNPs@CNC suspension was directly influenced to the presence of the formed AgNPs. The concentration of Ag^+^ plays a crucial role in the antibacterial activity. In Fig. 7c, T1–T3, T4–T6 and T7–T9 samples revealed that as the amount of silver nitrate added gradually decreases, the inhibition zone of *E. coli* and *S. aureus* decreases, respectively. Similarly, using extreme range analysis indicated that the concentrations of CNC, and the power of the microwave and the time of reaction have less influence on the antibacterial activity than concentrations of Ag^+^. With minimal use of silver nitrate, the T1 sample in the orthogonal assay showed excellent antibacterial activity against both *E. coli* and *S. aureus* in Fig. 7a and b. In addition, the width of the inhibition zone of the orthogonal assay samples basically corresponds to the intensity of the UV absorption peak at 420 nm. The respective width of inhibition zones for the T1 samples were found as 7.87 ± 0.34 mm and 7.33 ± 0.25 mm for *E. coli* and *S. aureus* (Fig. 7c), respectively. In this MIC assay, 1.5 mg mL^−1^ CNC and deionized water were selected as controls. The MIC of T1 sample for *E. coli* and *S. aureus* were both 10.6 μg mL^−1^, these values compare favourably with other experiments conducted with the commercial AgNPs.^9^ In the orthogonal assay design, although the T1 used the most AgNO_3_ to synthesize AgNPs, the amount of AgNO_3_ required by the T1 sample was only 0.03 mM (∼0.01 wt%). These antibacterial test results indicated that high levels of antibacterial activity could still be achieved with trace amounts of AgNO_3_.

## 4 Conclusions

In this study, we successfully synthesized AgNPs with CNC as the carrier *via* microwave reaction reduction. The UV/vis absorbance spectra showed a large amount of Ag in the suspension of AgNPs@CNC, especially sample T1, because the absorption peak at 420 nm is very high. The surface morphology and analysis of XRD indicated that there were a large number of small AgNPs on the CNC surface of the T1 sample. Structural characterization indicated that chemical bonds were not formed between AgNPs and CNC. This may be because the AgNPs were merely physically adsorbed on the CNC surface, as CNC has a large surface to volume ratio and negatively charged surface. The zeta potential analysis revealed T1 sample could be as stable as pure CNC suspension. In addition, TGA illustrated that the thermal stability of AgNPs@CNC was better than that of pure CNC, and this further illustrates that a large number of AgNPs were adsorbed on the CNC surface, which prevents the rapid decomposition of CNC at high temperatures. The antibacterial activity of T1 sample exhibited that only using 0.03 mM (∼0.01 wt%) AgNO_3_ to synthesize AgNPs@CNC could achieve good antibacterial activity. Therefore, this green, facile, and effective method reported in the present study may be promising way for the synthesis of AgNPs.

## Conflicts of interest

There are no conflicts to declare.

## Supplementary Material

RA-011-D0RA07198F-s001
