# Supplementary material for: Cellulose nanocrystal assisted trace silver nitrate to synthesize green silver nanocomposites with antibacterial activity
Source: RSC Adv. 2021 Jan 19;11(7):3808–15. doi: 10.1039/d0ra07198f (PMC8694241; doi:10.1039/d0ra07198f)
Supplement: RA-011-D0RA07198F-s001 [file RA-011-D0RA07198F-s001.pdf]

Electronic Supplementary Material (ESI) for RSC Advances.
This journal is © The Royal Society of Chemistry 2020

Cellulose nanocrystal assisted trace silver nitrate to synthesize green silver nanocomposites with antibacterial activity

Jiang Zhu,*[a] Tao Tang, [a] Chun-Yan Hu, [a] Wen-Cai Xiang, [a] Zhi-Qiang Chen, [a] Liu Luo, [a] He-Shan Yang [b] and Hong-Pan Liu [b]

[a] Chongqing Key Laboratory of Environmental Materials & Remediation Technologies, Chongqing University of Arts and Sciences, Yongchuan 402160, China.

[b] College of Chemistry and Environmental Engineering, Chongqing University of Arts and Sciences, Yongchuan 402160, China.

Table S1. L9 (3^4) The range analysis of the intensity of UV absorption peak at 420 nm.

#	CNC(mg/ml)	Ag+ ×10^2 (mM)	Power(w)	Time(s)	UV/vis absorbance at 410 nm
**T1**	1.5	3.00	320	200	0.80
**T2**	1.5	1.50	240	250	0.39
**T3**	1.5	0.75	160	300	0.28
**T4**	0.5	3.00	240	300	0.56
**T5**	0.5	1.50	160	200	0.29
**T6**	0.5	0.75	320	250	0.24
**T7**	0	3.00	160	250	0.38
**T8**	0	1.50	320	300	0.14
**T9**	0	0.75	240	200	0.10
**K1**	1.47	1.74	1.18	1.19	
**K2**	1.09	0.82	1.05	1.01	
**K3**	0.62	0.62	0.95	0.98	
**k1**	0.49	0.58	0.39	0.40	
**k2**	0.36	0.27	0.35	0.34	
**k3**	0.21	0.21	0.32	0.33	
**Range**	0.28	0.37	0.07	0.07	

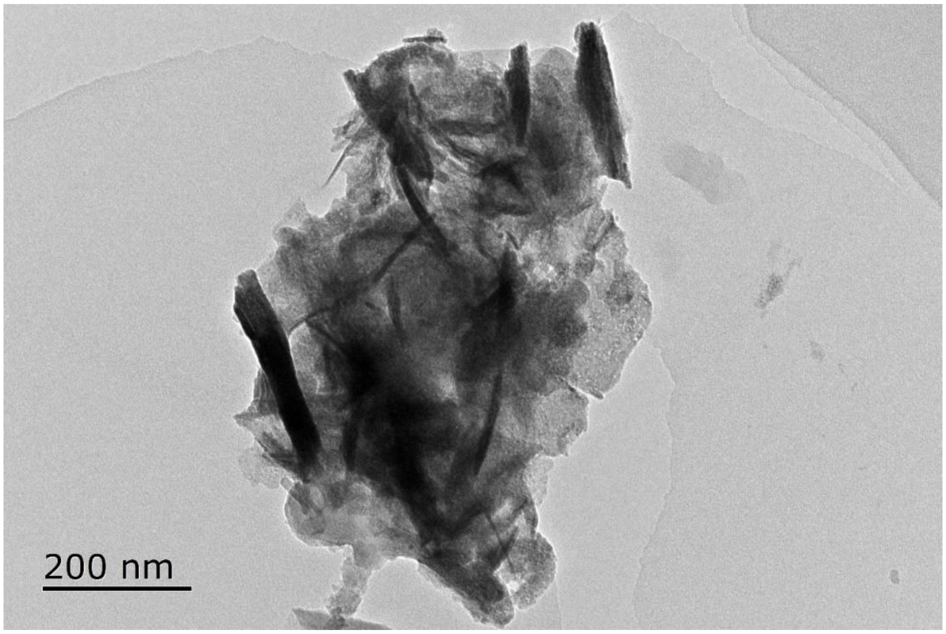

Figure S1. TEM image of pure CNC.

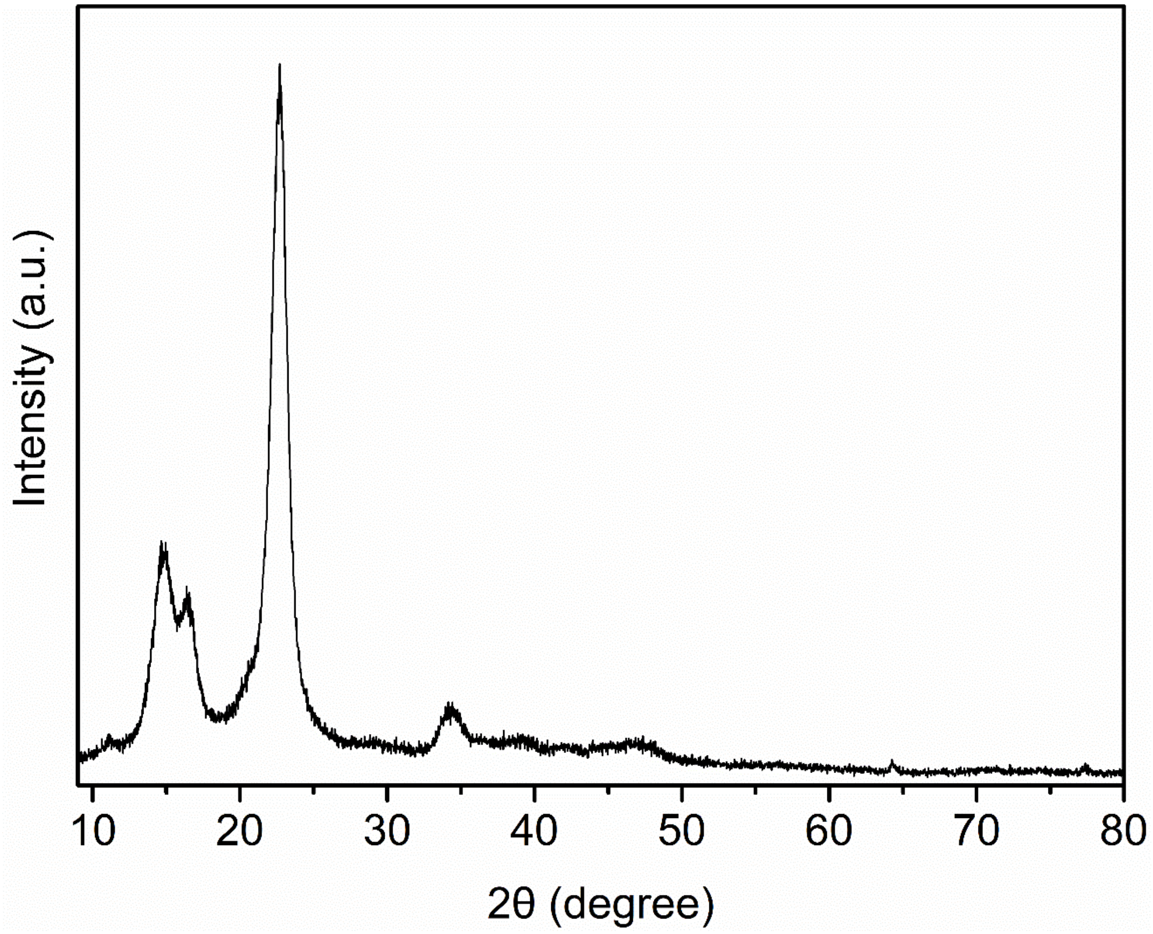

Figure S2. Wide-angle XRD patterns of the pure CNC.

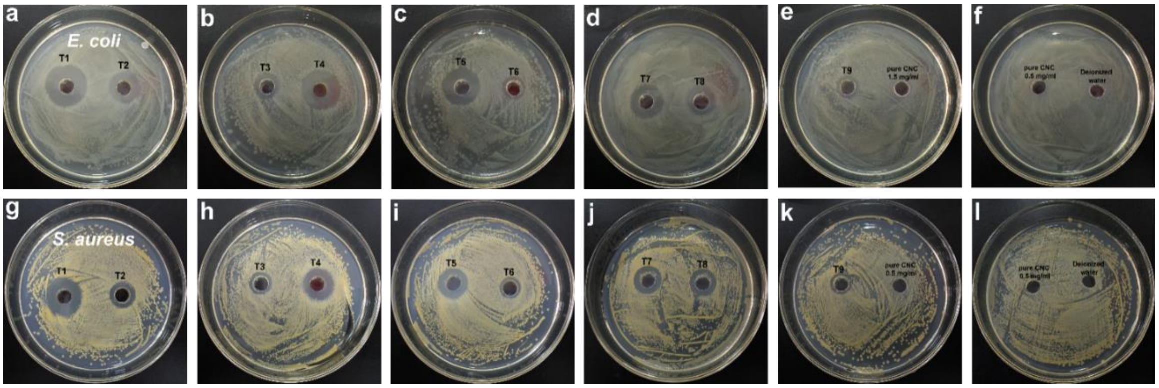

Figure S3. The photos of the antibacterial activity of AgNPs@CNC orthogonal array samples (a-l).
